# Reduced injury risk links sociality to survival in a group-living primate

**DOI:** 10.1016/j.isci.2022.105454

**Published:** 2022-10-31

**Authors:** Melissa A. Pavez-Fox, Clare M. Kimock, Nahiri Rivera-Barreto, Josue E. Negron-Del Valle, Daniel Phillips, Angelina Ruiz-Lambides, Noah Snyder-Mackler, James P. Higham, Erin R. Siracusa, Lauren J.N. Brent

**Affiliations:** 1 Centre for Research in Animal Behaviour, University of Exeter, Exeter EX4 4QG, United Kingdom; 2 Department of Anthropology, New York University, New York, NY 10003, USA; 3 Caribbean Primate Research Center, University of Puerto Rico, San Juan 00936-5067, Puerto Rico; 4 Center for Evolution and Medicine, Arizona State University, Temple, AZ 85281, USA; 5 Department of Human Behavior, Ecology and Culture, Max Planck Institute for Evolutionary Anthropology, Leipzig 04103, Germany; 6 School of Life Sciences, Arizona State University, Temple, AZ 85281, USA

**Keywords:** Biological Sciences, zoology, animal behavior

## Abstract

Sociality has been linked to a longer lifespan in many mammals, including humans. Yet, how sociality results in survival benefits remains unclear. Using 10 years of data and over 1,000 recorded injuries in rhesus macaques (*Macaca mulatta*), we tested two injury-related mechanisms by which social status and affiliative partners might influence survival. Injuries increased individual risk of death by 3-fold in this dataset. We found that sociality can affect individuals’ survival by reducing their risk of injury but had no effect on the probability of injured individuals dying. Both males and females of high social status (measured as female matrilineal rank and male group tenure) and females with more affiliative partners (estimated using the number of female relatives) experienced fewer injuries and thus were less likely to die. Collectively, our results offer rare insights into one mechanism that can mediate the well-known benefits of sociality on an individual’s fitness.

## Highlights

• Injuries increased the risk of dying in rhesus macaques
• Sociality reduced the risk of injury, thereby enhancing survival
• High-status animals and females with more affiliative partners were injured less
• However, once injured, sociality did not reduce the probability of dying

## Graphical abstract

**Figure:**
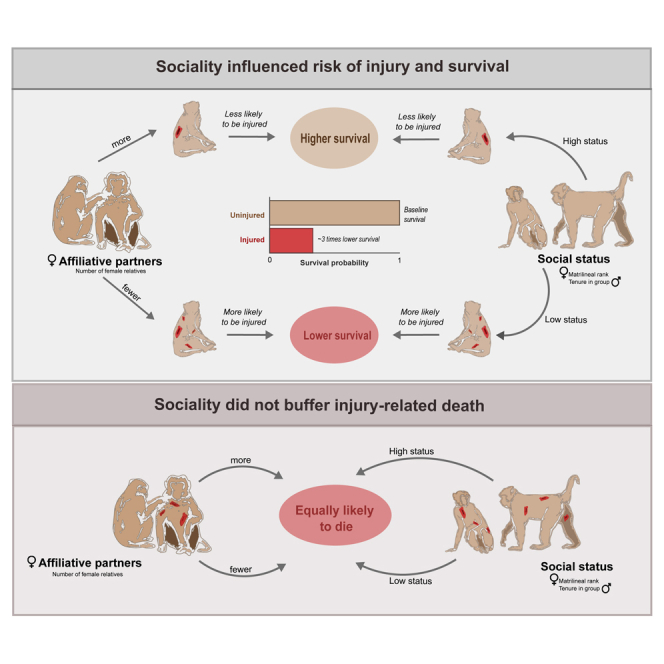

## Introduction

Uncovering the means by which sociality influences lifespan is of major interest to evolutionary biologists, social scientists, and biomedical researchers.^1^^,^^2^^,^^3^ Evidence from humans and other animals has provided increasing support for the benefits of affiliative social interactions on survival. The strength of social bonds^4^^,^^5^^,^^6^^,^^7^, the number of weak connections^8^, the number of associates^9^^,^^10^^,^^11^, the number of relatives in a group^12^, and the number of indirect connections^11^^,^^13^ all predict the lifespan of individuals; the general pattern being that those with more or stronger social relationships are the ones that live longer (however, see Blumstein et al.^14^ for the opposite effect in a facultative social mammal). Similarly, socioeconomic status in humans and social status in other animals are also robust predictors of mortality risk^5^^,^^12^^,^^15^^,^^16^^,^^17^^,^^18^, with lower-status individuals suffering a greater risk of death. But precisely how the social environment affects survival is less well understood.

One way for sociality to influence survival is by mitigating the costs of competition. Dominance hierarchies, for instance, are believed to have evolved to reduce direct costs associated with competition for resources^19^. Nevertheless, social hierarchies still usually entail disparities in resource access, with individuals higher in the hierarchy having priority access to food and mates at the expense of their subordinates^20^, who may still need to compete for resources. Affiliative partners can also help to reduce engagement in agonistic encounters by providing access to resources via cooperation and social tolerance^21^. For example, food sharing, cooperative feeding, and co-feeding have been described in several mammals, including some species of bats^22^, cetaceans^23^^,^^24^^,^^25^, monkeys^26^^,^^27^, and apes^21^. Having affiliative partners in the group can also be advantageous to deter physical aggression from conspecifics by providing agonistic support. For instance, affiliative interactions predict the formation of coalitions in male and female African wild dogs (*Lycaon pictus*)^28^, Camargue horses (*Equus caballus*)^29^, macaques (*Macaca spp.*)^30^^,^^31^, and chimpanzees (*Pan troglodytes*)^32^. Agonistic support has been widely documented in female-philopatric primate species where related females defend one another^33^^,^^34^^,^^35^. If social status or affiliative relationships reduce the chance of aggressive interactions, these components of sociality may directly enhance survival by allowing individuals to avoid costly outcomes, such as injuries.

In addition to mitigating the immediate costs of aggressive behaviors, sociality may also enhance survival through buffering mechanisms that influence an individual’s health. Differences in access to resources according to social status, for instance, may determine the general body condition and health of individuals. Low social status has been related to higher disease risk^16^, higher levels of inflammation^36^^,^^37^, reduced healing capacity^38^, and overall impaired health in several mammal species, including humans^2^^,^^39^^,^^40^. Affiliative partners, also, can be valuable resources that can contribute to better health by providing access to food^41^^,^^42^ and by reducing the burden of infections via hygienic behaviors (*i*.*e*., grooming)^43^^,^^44^. Better health status for high-ranking or socially integrated individuals may translate into higher chances of survival in the face of adversity, for example, by improving the chances of recovery following an injury.

Yet despite clear hypotheses for the potential mechanisms by which social status and affiliative relationships influence lifespan, there remains a lack of empirical evidence that these mechanisms affect survival. Several studies have shown associations between individual variation in sociality with markers of health and immunity^36^^,^^37^^,^^45^^,^^46^, yet the consequences of such differences on survival are unknown. Similarly, studies supporting a relationship between sociality and lifespan usually do not have the detailed physiological or health data required to test potential mechanisms connecting the two^1^. To fill this gap, we used a long-term dataset containing both survival data and detailed information on injuries in a free-living population of rhesus macaques to test whether sociality mitigates the costs of competition (*i*.*e*., injuries) and its consequences on survival.

**Figure 1:**
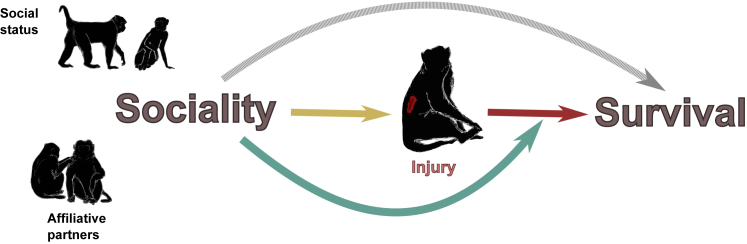
Injury-related mechanisms by which components of sociality (social status, affiliative partners) can influence survival A direct effect of sociality on survival (gray arrow) has been well established in mammals^4^^,^^5^^,^^7^^,^^10^^,^^11^, including studies in the Cayo Santiago population^8^^,^^12^. We explore mechanisms related to injury by which the relationship between sociality and survival might come about. According to the first mechanism, sociality influences the risk of injury (**yellow arrow**) and, therefore, survival (**red arrow**). According to the second mechanism (**green arrow**), sociality affects the survival trajectories of injured individuals.

We explored two injury-related mechanisms that can link sociality with survival. Specifically, we tested whether social status and/or affiliative relationships 1) influence the risk of being injured and/or 2) alter an individual’s survival trajectory after an injury (Figure 1). We did so using 10 years of injury data collected *ad libitum* together with demographic information from male and female rhesus macaques aged 4–29 years living on Cayo Santiago Island, Puerto Rico. Previous studies have shown the benefits of affiliative partners and social status on the survival probability of monkeys in this population^8^^,^^12^^,^^15^. Rhesus macaques live in multi-male multi-female despotic societies, where access to resources is strongly influenced by an individual’s position in the dominance hierarchy^47^. Predators are absent from Cayo Santiago, ensuring injuries are mostly the result of physical aggression between conspecifics. Rhesus macaques are seasonal breeders with a mating season that can last from 3 to 6 months. During the mating season, both affiliative and agonistic interactions are usually heightened^48^^,^^49^, and thus, important trade-offs between health, reproduction, and survival may occur at this time^50^^,^^51^.

Because our study hinged on the assumption that being injured was detrimental for survival in this population, we first tested whether injuries inflicted by conspecifics increased the probability of death in these animals (Figure 1; red arrow). To test if sociality influences the risk of injury (mechanism 1), we asked whether social status and the number of affiliative partners were associated with an individual’s injury risk (Figure 1; yellow arrow). Given the protective role of high social status and the importance of affiliative partners in deterring aggression^19^^,^^21^^,^^34^, we predicted that high-status individuals and those with more affiliative partners would have a lower risk of injury. To test if sociality can alter the impact of injuries on survival (mechanism 2), we asked if social status and the number of affiliative partners affected the survival trajectories of injured individuals (Figure 1; green arrow). As both social status and social integration can determine differences in health status that may affect recovery time^38^^,^^39^^,^^52^, we predicted that high-status animals and those with more affiliative partners would have a lower hazard of death from an injury than low-status individuals or those with fewer affiliative partners. Our results demonstrate that sociality plays an important role in mediating the risk of injury, offering one clear mechanistic link between sociality and survival in a group-living mammal.

## Results

### Effect of injuries on survival

**Figure 2:**
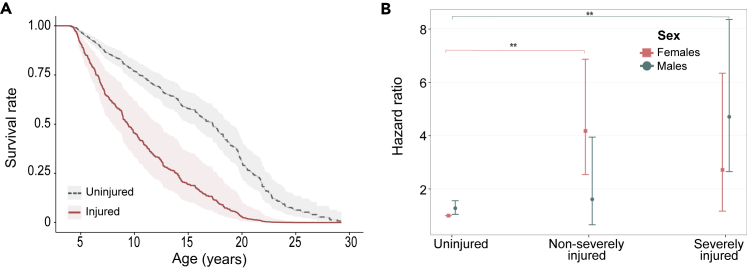
Effect of injuries on survival (A) Survival curves adjusted for covariates for injured and uninjured individuals. Injured individuals (red solid line, n = 571, 294 females, 277 males) had a near 3-fold increase in the probability of dying compared to uninjured animals (gray dashed line, *n* = 1030, 557 females, 473 males) (Hz = 1.07 ± 0.17, z = 6.24, p *<* 0.01, injuries (i) = 1041, deaths (d) = 443). Curves represent males during the mating season, but those for females were similar. Shaded areas represent standard errors. (B) Hazard ratios of death for females and males as a function of the severity of injuries. The main cause of injury-related death was from severe injuries in males (green circles, n uninjured = 473, n non-severely injured = 189, n severely injured = 251), and non-severe injuries in females (Pink squares, *n* uninjured = 557, *n* non-severely injured = 232, n severely injured = 147) (Hz severity∗sexM = 1.49 ± 0.72, z = 2.06, p = 0.039, i = 398, d = 107). Uninjured females represent the intercept and vertical bars depict the 95% confidence interval. Statistical significance in a post-hoc analysis is indicated by asterisks where ∗∗p *<* 0.01. All other pair-wise comparisons within and between sexes were not significant.

To quantify the extent to which injuries are associated with an individual’s survival, we used time-dependent mixed-effects cox models^53^^,^^54^. Animals that were injured were nearly three times more likely to die in the two months following an injury than animals that were not injured, independent of the reproductive season when the injury occurred (Figure 2A; Hazard [Hz] = 1.07 ± 0.17 [SEM], z = 6.24, p *<* 0.01, injuries [i] = 1041, deaths [d] = 443, N injured = 571, N uninjured = 1030; Table S1). The higher hazard of death associated with injuries was dependent on the severity of the injury and the sex of the animal (Hz severity∗sexM = 1.49 ± 0.72, z = 2.06, p = 0.039, i = 398, d = 107, N severely injured = 295; Figure 2B and Table S2). Males with severe injuries (e.g., broken bones, exposed organs, multiple wounds, or wounds in vital areas, see STAR Methods for details) were more likely to die than males who were uninjured (post hoc test: uninjured vs severely injured Hz = −1.27 ± 0.3, p *<* 0.01), but did not experience higher hazard than males with non-severe injuries (e.g., injuries in back, chest, limbs) (post hoc test: non-severely vs severely injured Hz = −1.06 ± 0.5, p = 0.11). Males with non-severe injuries also did not experience a higher mortality risk than uninjured males (post hoc test: non-severely injured vs uninjured Hz = 0.2 ± 0.46, p = 0.89). Females with non-severe injuries had higher mortality risk than uninjured females (post hoc test: non-severely injured vs uninjured Hz = 1.37 ± 0.26, p *<* 0.01), but did not differ in their hazard of dying when compared to females that were severely injured (post hoc test: non-severely vs severely injured Hz = 0.4 ± 0.5, p = 0.7). Severe injuries in females were not associated with a higher mortality risk than uninjured individuals (post hoc: uninjured vs severely injured Hz = −0.97 ± 0.44, p = 0.06). There were no significant sex differences in the hazard of death for severe injuries (females vs males Hz = −0.55 ± 0.5, p = 0.28) or for non-severe injuries (females vs males Hz = 0.91 ± 0.5, p = 0.07). In sum, when compared to uninjured animals, the main source of injury-related mortality for males comes from severe injuries, while in females it was associated with non-severe injuries.

### Mechanism #1: Sociality influences the risk of injury

#### Effect of social status on injury risk

To test if high-status animals were less likely to be injured or severely injured than low-status ones, we quantified the relationship between rank and injury risk separately for males and females using logistic models. We used proxies for social status as observations of agonistic interactions between pairs of animals (from which dominance rank is computed) were only available for a subset of subjects (1660 macaque-years). To maximize our statistical power, we decided to use the complete dataset (8459 macaque-years) and known proxies of social status instead. Specifically, we used group tenure for males^55^^,^^56^^,^^57^ and matrilineal rank for females^12^^,^^15^. Male rhesus macaques obtain dominance through queuing, whereby those males that have been in a group for longer are usually higher ranking^58^. Female rhesus macaques are philopatric and form maternally inherited stable linear dominance hierarchies whereby daughters occupy a rank just below their mothers^59^. Members of the same matriline tend to be adjacent to one another in the hierarchy; thus, the rank of an entire matriline can be used as a proxy for individual rank in social groups containing more than one matriline^15^. Both proxies had a strong association with social status obtained from behavioral observations in this same population (females: ß rankLow = −1.12 ± 0.085, p *<* 0.01; males: ß tenure = 0.67 ± 0.04, p *<* 0.01; Figure S2, details in STAR Methods).

**Figure 3:**
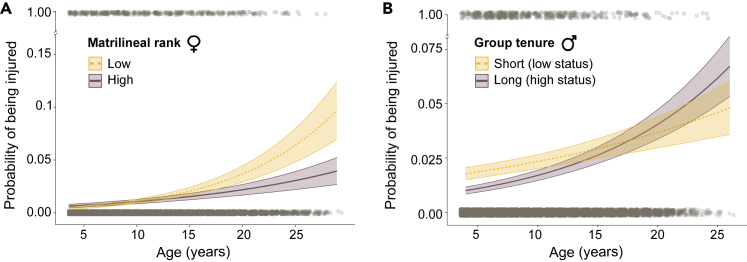
Effect of social status on injury risk (A) Injury risk for females as a function of matrilineal rank and age. Females from lower-ranking matrilines (yellow dashed line, *n* = 510, 237 injuries) had higher chances of being injured than females from higher-ranking matrilines (purple solid line, *n* = 325, 211 injuries), with increasing probabilities for older females (odds rankLow∗age = 0.23 ± 0.1, z = 2.43, p *<* 0.01). (B) Injury risk for males as a function of tenure length and age. For visualization, tenure length was categorized by selecting the 20th (273 days of tenure) and 80th (2029 days of tenure) percentiles depicting low status (yellow dashed line) and high status (purple solid line), respectively (*n* = 748, 536 injuries). Younger males from low status (shorter tenure) had higher injury risk than high-status (longer tenure) young males, yet the opposite occurred at later ages (odds tenure∗age = 0.1 ± 0.03, z = 3.04, p *<* 0.01). In both plots, shaded areas represent standard errors and gray dots the raw data used in the models (top: injured, bottom: uninjured).

We found that matrilineal rank in females had a strong effect on the likelihood of being injured, which was dependent on an individual’s age (log-odds rankLow∗age = 0.23 ± 0.09, z = 2.43, p = 0.015, i = 448, N = 817; Table S3). Low-ranking females had a higher probability of being injured than high-ranking females, but only at older ages (Figure 3A). Matrilineal rank had no relationship with the risk of severe injuries in females (log-odds rankLow = −0.17 ± 0.2, z = −0.86, p = 0.39, i = 135, N severely injured = 114; Table S4). In males, group tenure also had a strong effect on the probability of being injured that was dependent on age (log-odds tenure∗age = 0.1 ± 0.03, z = 3.04, p *<* 0.01, i = 536, N = 748; Table S5). Lower social status (i.e., shorter tenure) was associated with a higher incidence of injuries, but only at younger ages (Figure 3B). The same pattern was observed when we focused our analysis on severe injuries only (Figure S3A, log-odds tenure∗age = 0.11 ± 0.04, z = 2.54, p = 0.01, i = 245, N severely injured = 168; Table S6). Consistent with heightened male-male competition over females^48^ and with male harassment of females during the reproductive season^60^, we also found that injury risk increased for both males and females during the mating period compared to outside it, independent of their social status (injury: log-odds females = 0.85 ± 0.28, z = 3.03, p *<* 0.01; log-odds males = 1.21 ± 0.26, z = 4.57, p *<* 0.01; severe injury: log- odds females = 1.03 ± 0.26, z = 3.94, p *<* 0.01; log-odds males = 1.38 ± 0.26, z = 5.4, p *<* 0.01).

We used a post hoc path analysis to further confirm that social status can indirectly affect an individual’s survival by influencing their injury risk. We found that both matrilineal rank and tenure in a group (in interaction with age) can indirectly affect female and male survival by altering injury risk (Figure S4; females Fisher’s C statistic = 4.61, degrees of freedom [df] = 2, p = 0.329; males Fisher’s C statistic = 5.25, df = 2, p = 0.262; Tables S20–S26. Note that p *>* 0.05 in a path analysis indicates an acceptable fit of the model). In the case of males, an age-dependent direct effect of tenure on survival was also detected, even when accounting for injury status (Tables S19–S26), suggesting that there are other mechanisms linking tenure and survival independent of injury risk.

#### Effect of affiliative partners on injury risk

**Figure 4:**
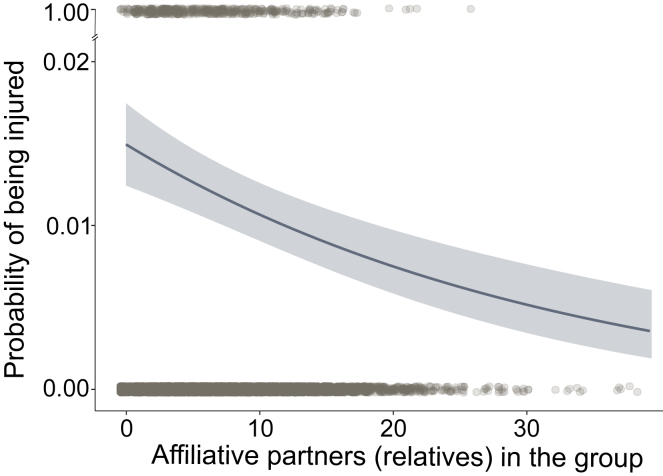
Effect of affiliative partners on injury risk The x-axis represents the number of adult female relatives (extended family, r ≥ 0.125) present in a female’s group (*n* = 851, injuries (i) = 491). Females with more relatives had lower chances of suffering from an injury than females with fewer relatives (odds = −0.13 ± 0.05, z = −2.5, p = 0.01, i = 491). Shaded areas represent standard errors, and gray dots the raw data used in the models (top: injured, bottom: uninjured).

To test whether animals with more affiliative partners were less likely to be injured or severely injured than those with fewer affiliative partners, we used logistic models. To support robust statistical analyses, we relied on a proxy for the number of affiliative partners that has been previously shown to influence survival in this population (i.e., the number of female relatives in the group)^12^. Female rhesus macaques have a strong bias toward forming partnerships with their maternal kin^61^, and the number of female relatives a female has in her group is positively correlated with network measures of how socially integrated she is^62^. Males, on the other hand, are the dispersing sex and have few kin in their new groups and so were excluded from this analysis. We found that the number of close relatives (relatedness coefficient [r] = 0.5, i.e., mother-daughters and full siblings) in a female’s group had a weak, but not significant, effect on her probability of being injured (log-odds = 0.09 ± 0.05, z = −1.89, p = 0.059, i = 491, N = 851; Table S7). However, the size of a female’s extended family (r ≥ 0.125, i.e., spanning three generations) was strongly associated with the likelihood of injury, with females experiencing a 47% reduction in the incidence of injuries for every one-standard-deviation (∼ 4 females) increase in their number of relatives (Figure 4A; log odds = −0.13 ± 0.05, z = −2.47, p = 0.014, i = 491, N = 851; Table S9). The incidence of severe injuries was not affected by the number of close relatives (log odds = −0.06 ± 0.09, z = −0.63, p = 0.53, i = 147, N severely injured = 123; Table S8), but the size of a female’s extended family was weakly associated with a reduced probability of being severely injured in periods outside the reproductive season (Figure S3B; log odds nkin∗season = 0.34 ± 0.17, z = 1.954, p = 0.05, i = 147, N severely injured = 123; Table S10).

Similar to the above, we used a post hoc path analysis and found that having more relatives in the group may indirectly affect a female’s survival by reducing her risk of being injured (Figure S5; Fisher’s C statistic = 5.09, df = 1, p = 0.078). We also found a strong direct effect of the number of relatives on a female’s survival that was independent of her injury status (Tables S19 and S27–S29). In other words, the survival benefit associated with having more female relatives in the group is also determined by other mechanisms not considered in our study.

### Mechanism #2: Sociality influences the survival of injured animals

#### Effect of social status on survival of injured animals

To assess whether social status or affiliative relationships buffer the detrimental effects of injuries on survival, we used time-dependent mixed-effects cox models. We found no evidence of a buffering effect of matrilineal rank on the survival of injured females (Hz injured∗rankLow = −0.13 ± 0.49, z = −0.27, p = 0.78, i = 448, d = 103, N = 278; Table S11) or group tenure on the survival of injured males (Hz injured∗tenure = 0.12 ± 0.22, z = 0.54, p = 0.59, i = 536, d = 97, N = 272; Table S13). Similarly, no buffering effect of matrilineal rank or group tenure on survival was observed in severely injured females (Hz injured∗rankLow = −0.4 ± 0.99, z = −0.41, p = 0.68, i = 135, d = 42, N severely injured = 114; Table S12) or males (Hz injured∗tenure = −0.15 ± 0.26, z = −0.57, p = 0.57, i = 245, d = 57, N severely injured = 168; Table S14), respectively.

#### Effect of affiliative partners on survival of injured animals

We found no evidence for a relationship between survival after an injury and the number of close relatives a female had available at the time (Hz injured∗nkin = −0.17 ± 0.21, z = −0.8, p = 0.42, i = 491, d = 114, N = 294; Table S15) or the current size of her extended family (Hz injured∗nkin = 0.11 ± 0.19, z = 0.6, p = 0.54, i = 491, d = 114, N = 294; Table S17). Similarly, the number of affiliative partners did not influence the survival of severely injured females (Hz close kin = −0.6 ± 0.5, z = −1.21, p = 0.225; Hz extended family = −0.04 ± 0.37, z = −0.11, p = 0.91; i = 147, d = 45, N severely injured = 123; Tables S16 and S18).

## Discussion

Taken together, our results suggest that different components of the social environment can modulate the risk of suffering an injury and, therefore, the hazard of death. We found that high social status was associated with a lower injury risk for specific periods of males’ and females’ lives and that a female’s affiliative partners may help to prevent injuries. In contrast to previous research showing that individuals with higher social status had faster healing rates^38^, we found that none of the measures of sociality we analyzed affected the survival trajectories of injured animals.

Injured individuals were substantively more likely to die than uninjured animals, demonstrating the high costs of physical aggression for macaques in this population and the relatively short period (i.e., two months) in which injuries can affect an individual’s fitness. Exactly how injuries affect the health of individuals and lead to death remains an open question. On the one hand, injuries could have a direct effect on survival as a result of lethal attacks. Lethal attacks seem to be common in great apes^63^ and, although they might occur less often, have been documented in other mammals^64^, including rhesus macaques from the Cayo Santiago population^65^. On the other hand, injuries can affect survival indirectly, for example, by impairing locomotion, which may lead to problems in acquiring resources to sustain wound healing or basic energetic demands, or also by exposing damaged tissue to infectious diseases^66^^,^^67^. But regardless of how exactly death comes about, our results provide evidence that injuries from conspecifics can be fatal.

Males experienced a greater hazard of death only from severe injuries, which might reflect trade-offs between the energy allocated for reproduction versus immunity^38^^,^^66^ and/or the areas of the body affected. During the reproductive season, the probability of being severely injured was substantially higher for both sexes. During this period, males may particularly be immunocompromised due to the high amount of energy and resources required to sustain the effort associated with mating^50^^,^^68^ which can impair injury recovery, especially when suffering multiple injuries at once or injuries in very vulnerable areas, such as the genitalia (Figure S6). Females, on the other hand, seemed to be more affected by non-severe injuries than severe ones. Sample size may explain this result as females were less often severely injured than males (*n* females = 147, *n* males = 251), and so, our ability to detect effects of severe injuries in females was more limited. We also cannot disregard that our definition of severe injuries might not apply equally to males and females; what constitutes a non-severe injury in males might actually be severe for females or vice versa. Future studies with a large enough sample size to look at sex differences in the trade-offs between immunity and reproduction in the context of injury are required to determine how severe injuries affect survival in this and other populations of group-living animals.

We found support for one of the hypothesized mechanisms linking sociality to survival, whereby sociality reduces an individual’s risk of injury. High-social-status animals (high matrilineal rank in females and longer group tenure in males) were less likely to be injured than lower-status animals during specific periods of their life. Females with more affiliative partners (i.e., more relatives) were less likely to be injured than less integrated females and, thus, experienced a lower hazard of death.

Our results linking social status to reduced risk of injury are consistent with the skewed access to resources in systems with clear linear dominance hierarchies^20^. Although we do not have information on the context in which injuries occurred, our results suggest that high-status individuals may not need to engage in costly aggression for food or mates as often, in contrast to low-status animals who must gain access through contests. Our results suggest that low-status individuals experience a greater hazard of death than high-status individuals because they have an enhanced injury risk, which was supported by our path analysis.

Our finding that matrilineal rank influenced the risk of injury in older but not younger females may be because at younger ages, females’ relative positions in the dominance hierarchy have yet to be fully established^69^. Also, by using matrilineal rank as a proxy, we might not have enough resolution in the dominance relationships between females within a matriline or even between low-ranking matrilines to be able to capture rank-based differences in injury risk, especially at younger ages when the occurrence of injuries is lower. Further, we showed that low-status males were more likely to be injured than high-status males, but only at younger ages. The only way for a young male to have high status (i.e., long group tenure) is for him to have not yet dispersed from his natal group. These young, high-status males may get protection benefits from their maternal relatives, which would not be the case for young males recently joining a new group. Indeed, rhesus macaque males that delay their dispersal are usually the sons of the high-ranking female and by extension have the privileges of their mother’s social status^70^^,^^71^, which may help them to buffer their risk of injury. Finally, the lack of differences in injury risk with social status in prime-age and older males could be attributed to reduced benefits associated with tenure in the context of mating competition^72^, which is expected to be the main driver of contests between males once they reach their peak in sexual maturity.

Previous studies in matrilineally structured species, where most affiliative relationships are between female relatives, have shown that females commonly engage in agonistic encounters to support and protect their kin^33^^,^^34^^,^^35^, even when confronting higher-status individuals^73^. In line with these findings, our results suggest that having more relatives available may provide a numerical advantage to deter physical aggression. Interestingly, we also found that having more female relatives was associated to reduced risk of severe injury during the non-reproductive season, suggesting that more affiliative partners might be especially advantageous to deter fierce aggression in contexts outside of direct competition for mates. However, other mechanisms, such as social tolerance when accessing resources^21^, could also explain fewer injuries in the presence of more affiliative partners. For instance, females might be less likely to engage in aggressive interactions when feeding with relatives or be more willing to share a feeding spot with kin than with non-kin. Finally, our results suggest that both the number of close and extended kin that a female has contribute to reduce her risk of being injured, even though when looking at the number of close kin alone we might not have had enough variation to detect robust effects (Figure S7). A path analysis supported the effect of social integration on injury risk as an indirect route to female survival. But it is also important to note that the number of relatives also had a strong, direct effect on survival. Combined, these results suggest that although social relationships might have evolved to help individuals avoid potentially fatal injuries from their conspecifics, it is unlikely to be their sole ultimate function. Other mechanisms not included in this study should be considered as alternative and/or complementary routes between social relationships and survival. In other words, the ultimate function of within-group social relationships is likely to be multifaceted, including—but not limited to—injury prevention.

We found no support for the second hypothesized mechanism linking sociality to survival. None of the measures of sociality we analyzed were related to an individual’s survival trajectory following injury. Despite a vast body of literature supporting differences in health and immunity between individuals of different social status^36^^,^^39^^,^^45^, we found no evidence for an effect of social status on the survival trajectories of injured animals. These findings contrast with a previous study on wild baboons where high-status males had faster healing rates than lower-status males^38^. Although we did not quantify differences in healing times with social status, our results suggest that the probability of recovering from an injury was not influenced by an animal’s position in the dominance hierarchy. These differences might be explained in part by differences in features of the two study systems. Animals on Cayo Santiago are provisioned with food daily, and access to the nutrients needed to support immune function might not be as skewed as they are in the wild^46^. Notwithstanding, high social status in both systems has been associated with elevated levels of glucocorticoids and androgens^50^^,^^74^^,^^75^, which are well-known immune suppressors. Overall, this might suggest that in the Cayo Santiago population, unlike the baboons, the benefits of being of high status may not outweigh the costs in terms of helping to promote injury recovery.

We also found, contrary to our predictions, that the benefits associated with affiliative partners, such as feeding tolerance^76^^,^^77^ and social hygienic behaviors^43^^,^^44^, seem not to have helped females to cope with the detrimental effect of injuries on survival. It is possible that social hygienic behavior, such as the removal of ectoparasites by grooming, has long-term health benefits but does nothing to enhance the short-term immune response required to heal damaged tissue^78^. Additionally, grooming wounded areas may, in fact, be detrimental to the healing process as it could lead to the removal of protective scabs^44^. This could be one reason why females with more affiliative partners, who are presumed to receive more grooming and to have more access to food via social tolerance, did not have improved survival trajectories after an injury. Previous research on this population has shown that the number of close relatives and the size of a female’s extended family are associated with increased survival probability^8^^,^^12^. The results of the current study suggest this relationship does not come about because of the reduced risk of death from injury. Further research is needed to elucidate to what extent other mechanisms involving health differences (e.g., disease susceptibility) play a role in the benefits of social partners in the survival of females in this population.

In sum, our study provides evidence for a mechanism linking sociality to lifespan. Growing literature has supported a strong relationship between the social environment and survival in many mammal species^2^, but the ultimate function of some components of sociality, such as social relationships, remains unclear^79^. Although sociality has been demonstrated to enhance health and immunity^36^^,^^45^^,^^46^, here we showed that these benefits did not translate to an improved ability to cope with the risk of death from injuries. Instead, we found that sociality plays an important role in preventing individuals from suffering injuries that would likely lead to death. Given how rare injuries are in this population, we do not expect that this is the only mechanism linking sociality to survival. Other mechanisms may include sociality-mediated differences in components of health related to disease susceptibility. In wild animal populations, social partners may also help with predator detection^80^, predator mobbing^81^, finding food sources^82^, thermoregulation^83^, among other possibilities. Nevertheless, here we provide rare empirical evidence for an ultimate function of social relationships, showing one mechanism by which high status and socially integrated individuals live longer. Demonstrating the relative importance of different mechanisms linking sociality and survival will be challenging but a crucial goal of future research. Our study highlights the essential role that long-term datasets that combine both demographic and health data will play in meeting this challenge.

### Limitations of the study

This study relied on proxies for sociality instead of direct behavioral observations due to limitations in the sample size of matching data for injuries and animals with behavioral information. Additionally, we did not have direct evidence of the contexts in which injuries occurred; thus, the means by which sociality might reduce injury risk were somewhat speculative. Future studies should build on our findings by using direct behavioral observations in a large sample of individuals with paired injury data to explore refined ego-networks characteristics and to expand these results to affiliative relationships of males and unrelated females.

## STAR★Methods

### Key resources table

**Table:** 

REAGENT or RESOURCE	SOURCE	IDENTIFIER
**Deposited data**		

Cleaned data	Mendeley Data	https://doi.org/10.17632/95xxf29472.1
Code	GitHub	https://github.com/MPavFox/Injury-and-survival

**Experimental models: Organisms/strains**		

Free-ranging rhesus macaques: 851 females and 750 males	Not Applicable	Not Applicable

**Software and algorithms**		

R v4.1.3	R Core Team 2022	https://cran.r-project.org/
R package mice	Van Buuren & Groothuis-Oudshoorn 2011	https://cran.r-project.org/web/packages/mice/index.html
R package survival	Therneau 2022	https://cran.r-project.org/web/packages/survival/index.html
R package coxme	Therneau 2018	https://cran.r-project.org/web/packages/coxme/index.html
R package lme4	Bates et al. 2015	https://cran.r-project.org/web/packages/lme4/index.html
R package emmeans	Lenth et al. 2022	https://cran.r-project.org/web/packages/emmeans/index.html
R package survminer	Kassambara et al. 2021	https://cran.r-project.org/web/packages/survminer/index.html
R package effects	Fox 2003	https://cran.r-project.org/web/packages/effects/index.html
R package ggplot2	Wickham 2016	https://cran.r-project.org/web/packages/ggplot2/index.html
R package sjPlot	Ludecke 2022	https://cran.r-project.org/web/packages/sjPlot/index.html
R package car	Fox & Weisberg 2019	https://cran.r-project.org/web/packages/car/index.html
R package ggdag	Barret 2022	https://cran.r-project.org/web/packages/ggdag/index.html
Inkscape v1.0.1	Inkscape project	https://inkscape.org

### Resource availability

#### Lead contact

Further information should be directed to and will be fulfilled by the lead contact, Melissa A. Pavez-Fox (melissa.pavez.fox@gmail.com).

#### Materials availability

This study did not generate new unique reagents.

### Experimental model and subjects details

#### Subjects

Subjects were 851 female and 750 male free-ranging rhesus macaques (*Macaca mulatta*) living in the Cayo Santiago field station, Puerto Rico. Females were between 4 and 28 years of age, and males were between 4 and 22 years of age.

#### Ethical guidelines

This research complied with protocols approved by the Institutional Animal Care and Use Committee (IACUC) of the University of Puerto Rico (protocol no. A6850108) and by the University of Exeter School of Psychology’s Ethics Committee. The CPRC’s Animal Care and Use Program is evaluated and approved by the IACUC.

#### Method details

We studied a population of free-ranging rhesus macaques on the island of Cayo Santiago in Puerto Rico. The island is home to a population of ∼ 1800 individuals living in 6–10 mixed sex naturally formed social groups. The field station is managed by the Caribbean Primate Research Center (CPRC), who monitor the population daily, and maintain the long-term (*>* 75 years) demographic database including data on births, deaths, social group membership for all animals and a genetic parentage database for animals born after 1992.^84^ Animals have *ad-libitum* access to food and water, the island is predator-free and there is no regular medical intervention for sick or wounded individuals. We focused on all subadult and adult females and males between 4 and 28 years of age that were alive between the years 2010 and 2020, a period for which records on injuries exist (see below for details on how injury data was collected). In this study we included data on 571 injured individuals (294 females, 277 males) and 1030 uninjured individuals (557 females, 473 males). From these animals, 342 (85 injured, 258 uninjured) were removed from the population by the CPRC for population control purposes.^85^ For all individuals, birth dates were known within a few days. Removal dates were known for all removed individuals. Dispersal from the island almost never occurs, therefore death dates were also known within a precision of a few days.

#### Observation of injuries

From 2010 to 2020 CPRC staff collected ad-libitum observations on the incidence and recovery of injuries, during the daily monitoring of social groups for demographic purposes. Monkeys were individually recognized based on their identity tattoos located on their chest and leg. Whenever a staff member noticed a wounded animal or an animal displaying signs of injury (*e*.*g*., bleeding, limping), they recorded the animal ID, type of injury and additional details on the general state of the animal (*e*.*g*., by evidence of weight loss or poor physical condition). If there was a visible wound, observers additionally recorded the area of the body affected, if it was a recent or old wound based on the presence of scars, and whenever possible, an estimate of its size. Observers updated the records every time they encountered the injured animal during their daily census routine with an average update time for an injured individual across the 10 years of 42.17 days. In total, 1137 injury events were observed with an average of 107.6 ± 63.5 per year. Here, we included all the records of injuries that were considered non-ambiguous (i.e., those with visible damage to the skin) including bites, scratches, cuts and abrasions along with other clearly observable injuries such as fractures and exposed organs. As there are no predators on the island and no-human intervention during the data collection, we are confident that most of the injuries included here, if not all, are the result of conspecific aggression. Our final sample consisted of 1041 injuries collected from September 2010 to April 2020. We classified these injuries based on their degree of severity, where severe injuries were those involving broken bones, exposed organs, multiple wounds and any wound in vital areas, including head, neck, abdomen or genitalia (*n* = 398). All other injuries were considered non-severe (*n* = 643) (Figure S6 for details).

#### Measures of sociality

Given that observations of social interactions were only available for a subset of our subjects, we used established proxies of sociality to maximize our statistical power (n injuries = 292 for animals with behavior vs 1041 for full dataset). As proxy of social status, we used group tenure in males^55^^,^^56^^,^^57^ and matrilineal rank in females.^12^^,^^15^ We determined tenure length using information on monthly social group membership. Group tenure length was computed as the time (in days) a male has been observed in his current group at the date of interest (current date minus date of dispersal). If a male had not yet dispersed and remained in his natal group, we computed group tenure since their birth date. If a male died or was removed from the population before the end of the period of interest, we computed group tenure up to that point. We established tenure length for all the males in our dataset (*n* = 750, *n* injuries = 550). However, 67 of those males had periods where they were observed living outside a social group (*i*.*e*., they were “extra-group”). These specific periods when group tenure could not be computed were dealt differently depending on the analysis in question and we discuss this on a case-by-case basis below.

We determined matrilineal rank by first identifying the number of matrilines in each of the behavioral groups with injury records (*n* = 10 groups). A matriline was defined as all the descendants of a female founder of the group. Because rank is a relative measure, we disregarded groups with only one founder female in the group, i.e., one matriline (*n* = 4 groups disregarded). For all of the groups with 2 or more matrilines, we had behavioral observations for at least one of the ten years of study, allowing us to use data on dyadic agonistic interactions to identify the alpha female in each group. We identified only one matriline per group as “high-ranking”, the one containing the alpha female, while all the others in the group were classed as “low-ranking”. A female’s matrilineal rank is determined by her family lineage and does not change across her lifespan unless she changed group membership due to rare fission events. For the small number of cases where a fission occurred, we determined a female’s new matrilineal rank based on their relatedness to the alpha female in the newly established group. We established matrilineal rank for 817 unique females (325 classed as high-ranking and 510 as low-ranking, *n* injuries = 448).

To confirm that group tenure and matrilineal rank were appropriate proxies for social status we tested at the association between dominance ranks computed from animals with known social status based on agonistic interactions and our proxies. Social status from behavioral observations was determined as the number of group members of the same sex that the focal outranked, where 100% represents the highest ranking individual.^86^To determine the strength of association between our proxies and social status, we ran linear regressions where the dependent variable was social status (% outranked) and the predictor, our proxy (matrilineal rank or tenure in the group) while accounting for repeated measures per individual for those animals that had a behavioral observation for more than one year. We found that both matrilineal rank (ß rankLow = −1.12, p *<* 0.01) and tenure in a group (ß tenure = 0.67, p *<* 0.01) were strongly associated to social status in females and males, respectively (Figure S2).

As above, we only had data on affiliative interactions for a subset of our subjects. Therefore, to maximize our sample size we followed a previous study^12^ and used the number of female relatives (4 years and older) that were present in a female’s social group as a proxy for social capital. Female rhesus macaques preferentially interact with their female kin compared to non-kin individuals,^59^ thus those with greater number of relatives are expected to have more opportunities for social support. A previous study on this same population has shown that this proxy is positively associated with network measures of social integration.^62^ We limited this approach to females as males, being the dispersing sex, often have very few close kin in their new groups, and might not be able to recognise unfamiliar kin.^87^ Using the Cayo genetic pedigree database we computed the number of close kin (*r* = 0.5) and extended family (r ≥ 0.125) for all injured and uninjured females in our dataset (*n* = 851, *n* injuries = 491). We decided to test these two levels of relatedness as the first represents the strongest kin-bias (*i*.*e*., mother-daughter or full sisters) and the second the lowest threshold for kin bias in affiliative interactions for rhesus macaques.^88^ To test whether both of our proxies in females (matrilineal rank and number of relatives in the group) were measuring similar biological effects, we tested for an association between them while controlling for individual ID and group membership (both as random effects). We found that matrilineal rank did not predict the number of close adult female relatives (ß rankLow = −0.05, p = 0.324), nor the number of extended adult female family members (ß rankLow = −0.02, p = 0.738).

### Quantification and statistical analysis

For all of the statistical analyses we defined a two-month time window (hereafter, bimonthly interval) as the period from which the injury status could transition from injured to not injured based on the average update time for an injured animal (*i*.*e*., average time between two consecutive records) and the computed average healing time (details Data S1). Thus, all variables were evaluated on a bimonthly basis (*i*.*e*., each row in the dataset represents a two-month interval). For each of the questions, we ran two models, one that included injury status based on all injuries (model 1) and other that included injury status for severe injuries only (model 2). Statistical significance was based on p-values, where p = 0.05 was considered a weak effect, p *<* 0.05 a moderate effect and p *<* 0.01 a strong effect.^89^

#### Effect of injuries on survival

To establish the effect of injuries on survival we used time-dependent Cox proportional hazard (PH) models.^53^ For the analyses, we used the whole dataset (*n* = 1061), including injured and uninjured animals from both sexes. Animals that were removed from the population or that were still alive at the end of the study period were censored (Figure S1 for details on sample size). The predictor of interest was the injury status (*i*.*e*., all injuries or severe injuries) along with other relevant variables that may influence survival probability, such as reproductive season (*i*.*e*., mating vs non-mating) and sex. Age was accounted for implicitly in the models. Additionally, we included random effects for the specific bimonthly interval within the study period to control for potential mortality sources at the population level, individual identity to account for repeated measures and social group to account for potential confounders associated with group membership such as differences in group size (Tables S1 and S2). To determine the bimonthly interval we divided the whole study period (10 years) into intervals of two months- ranging from 1 to 58 - where 1 represents the first two months since September 2010. We tested for interaction effects among our predictors and only retained them if statistically significant to avoid issues of overfitting.

#### Mechanism #1: Sociality influences the risk of injury

To assess the effect of social status and the number of affiliative partners on the risk of injuries, we used generalized linear mixed models with binomial distribution (logit models). In all the models we asked whether our measures of sociality influenced the probability of being injured in a given bimonthly interval. To test if high status animals were less likely to be injured compared to low status ones, we ran the analyses separately for each sex (*n* females = 827, *n* males = 750). For males, social status was estimated from group tenure computed up to the end of each bimonthly interval. Bimonthly intervals where males were extra-group and so group tenure could not be computed, were excluded. For females, we used matrilineal rank, which remains constant across the lifespan and, thus, remained the same in every interval unless they split from a previous group. To test if animals with more affiliative partners were less likely to be injured compared to animals with social partners we used only females (*n* = 851), fitting separate models for the two thresholds of relatedness (close kin and extended family). The number of relatives present in a group was computed for each bimonthly interval. We modeled injury status as a function of social status or number of affiliative partners, while controlling for age and reproductive season. As group tenure and age could be correlated, we checked for collinearity between these predictors using the variance inflation factor (VIF), but no correlation was found (VIF = 1.01). Random effects were included for individual ID to account for repeated measures, social group and for the specific bimonthly interval within the study period. We z-scored continuous variables to help convergence and tested interaction terms among all our predictors, which were retained if significant (Tables S3–S10).

#### Mechanism #2: Sociality influences the survival of injured animals

To examine the effect of sociality (social status and number of affiliative partners) on the survival of injured animals we used time-dependent cox PH models. As before, we tested for an effect of social status on survival in separate models for males and females and examined only females to test the effect of affiliative partners on survival post-injury. In all the models the predictor of interest was specified by an interaction term between injury status and the sociality measure. Variables were evaluated on a bimonthly basis with a time-dependent covariate for reproductive season. Random effects were included for individual ID, social group and bimonthly interval. As some bimonthly intervals had missing information for group tenure, we ran two models for males; a complete case analysis and a model using mean-matching multiple imputation with 20 iterations to fill the missing data,^90^^,^^91^ yet the estimates were identical between both procedures. Given that the main predictor was an interaction term, we did not attempt to fit other interactions (Tables S11–S18).

#### Post-hoc confirmatory path analysis

To further confirm our findings that sociality significantly influences survival by reducing risk of injury we ran a confirmatory path analyses.^92^ Briefly, this analysis allow us to test the causal association between multiple observed variables when the data has a hierarchical structure with different distributions (generalized linear mixed models) and assuming that the causal explanation does not involve unmeasured variables. Our first step in path analysis was to establish Directed Acyclic Graphs (DAG) showing the potential causal relationships between the variables in the models (Figures S4 and S5). All the variables not connected by an arrow in the DAGs are assumed to be independent in the model. Path coefficients were obtained from models predicting the endogenous variables (*i*.*e*., social capital, injury risk and survival). Then, we tested the independence of these variables; the estimate for each pairwise comparison is expected to be zero (H0) and have non-significant probability (details on claims of independence Table S19). We decided to only test claims of independence that are biologically meaningful. For example, testing if age or rank are independent of season does not add useful information to the model and may result in higher rates of type II error. Given that we had interaction terms, we did not determine the independence between the interaction variable (age x social status) and their main effects as their error terms were correlated. Finally, to determine goodness of fit we extracted the probabilities from the main predictors in the independence claims, which were used to compute the *Fisher C’statistic*. If the *C-statistic* absolute value is above 1.96 (p *>* 0.05), then we fail to reject the model as the data-generating process or in other words, the observed data does not significantly differ from the expected causal model.

All statistical analyses were done in R version 4.1.3^93^ using the survival,^94^ coxme,^95^ mice,^90^ emmeans^96^ and lme4^97^ packages. We checked for model assumptions using survival and car^98^ R packages. If models did not converge, we extended the maximum number of iterations to 200,000. In case of singular fit (*i*.*e*., overfitting issues), we excluded the random effect for group. Plots were done using the survminer,^99^ effects,^100^ sjPlot,^101^ ggdag,^102^ ggplot2^103^ R packages. Aesthetics of the plots were adjusted in Inkscape v1.0.1.^104^

## Data Availability

• Data have been deposited at Mendeley Data and are publicly available as of the date of publication. DOI is listed in the key resources table. • All original code has been deposited on GitHub and is publicly available as of the date of publication. The link is listed in the key resources table. • Any additional information required to reanalyze the data reported in this paper is available from the lead contact upon request.
