# Supplementary material for: Reduced injury risk links sociality to survival in a group-living primate
Source: iScience. 2022 Oct 31;25(11):105454. doi: 10.1016/j.isci.2022.105454 (PMC9667306; doi:10.1016/j.isci.2022.105454)
Supplement: Document S1. Figures S1–S7 [file mmc1.pdf]

iScience, Volume *25*

Supplemental information

Reduced injury risk links sociality to survival in a group-living primate

Melissa A. Pavez-Fox, Clare M. Kimock, Nahiri Rivera-Barreto, Josue E. Negron-Del Valle, Daniel Phillips, Angelina Ruiz-Lambides, Noah Snyder-Mackler, James P. Higham, Erin R. Siracusa, and Lauren J.N. Brent

Supplemental Information

Supplementary Figures

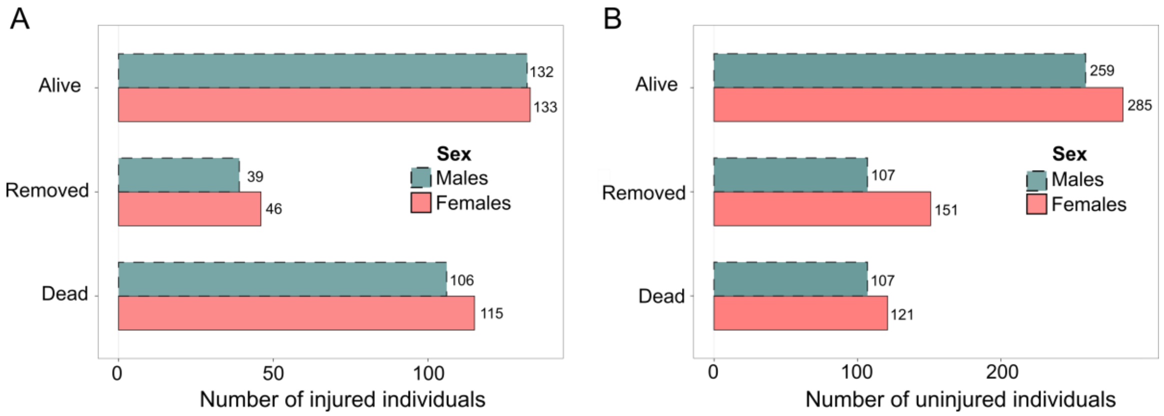

Figure S1: Sample sizes of injured and uninjured individuals by sex [Related to STAR Methods]. **A)** Number of injured males (green dashed contour) and females (pink solid contour) that were removed (*n* = 85) or died (*n* = 221) during the 10-years study period (2010 to 2020), or that were alive by the end of it (*n* = 265). **B)** Number of uninjured males and females that were removed (*n* = 258) or died (*n* = 228) during the period of study, or that were alive when it finished (*n* = 544).

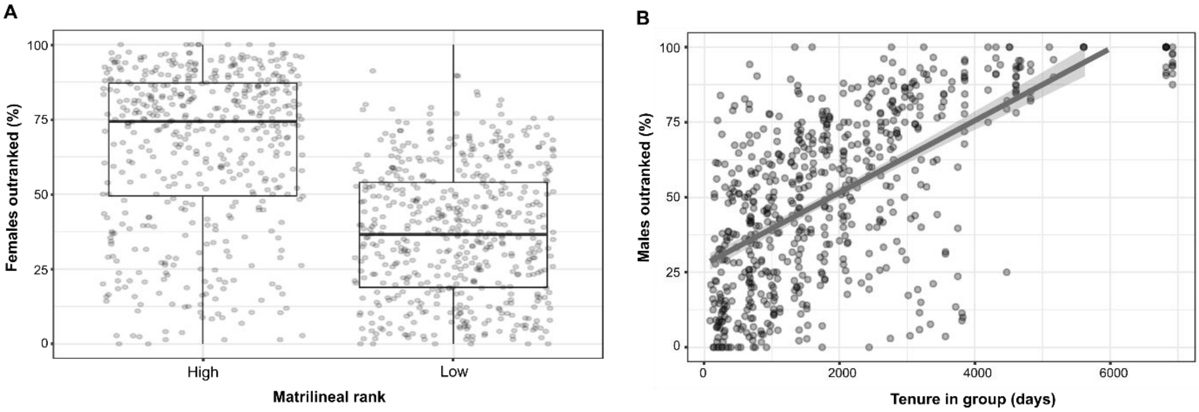

Figure S2: Association between social status obtained from behavioural observations and proxies used in the study [Related to STAR Methods]. **A)** The matrilineal rank of a female significantly predicted the percentage of females that she outranked in a group (100% = highest ranking animal) (estimate rankLow = -1.12, $p < 0.01$). Cases where the behavioral and maternal rank of a female do not match are due to rare occasions such as evictions from group members that might eventually return to their natal group. **B)** The tenure of a male in a group strongly predicted the percentage of males in the group that he outranked (estimate tenure = 0.67, $p < 0.01$).

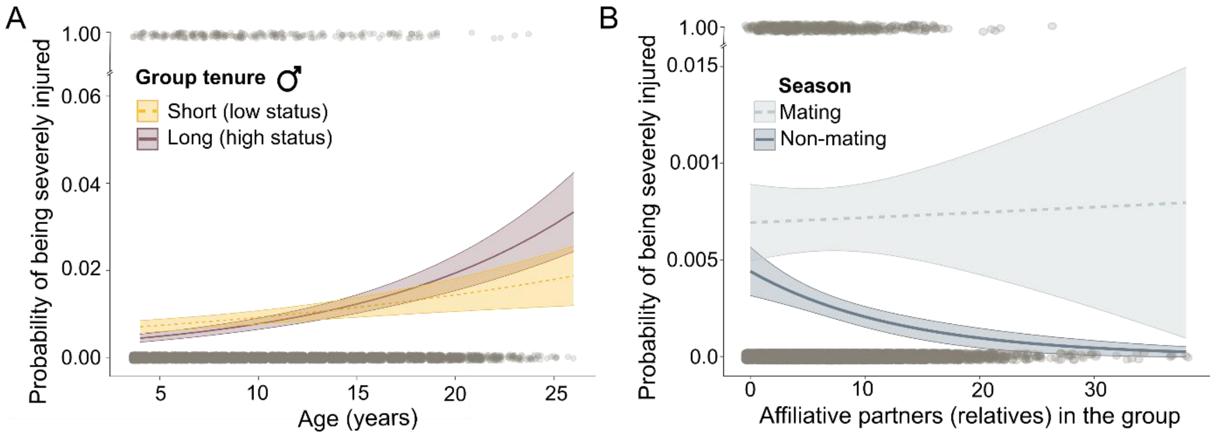

Figure S3: Predicted risk of severe injury as a function of sociality [Related to Figure 3 and Figure 4]**. A)** Injury risk for males as a function of tenure in group and age. Social status was categorized by selecting the 20th (273 days of tenure) and 80th (2029 days of tenure) percentiles depicting low status (yellow dashed line) and high status (purple solid line), respectively (n = 748, 245 severe injuries). Low status younger males had higher probability of being severely injured than high status young males (Odds tenure*age = 0.12 ± 0.04, p < 0.01). **B)** Injury risk for females as a function of the number of female relatives ($r \geq 0.125$) in the group and reproductive season. Females had a trend to have reduced risk of severe injury for every increase in female relatives in the group, but only during the non-reproductive season (blue solid line, reproductive season = dashed grey line) (Odds relatives*season = 0.34 ± 0.1, p = 0.051, n = 851, 147 severe injuries). Shaded areas represent standard errors and grey dots, the raw data used in the models (top: injured, bottom: uninjured).

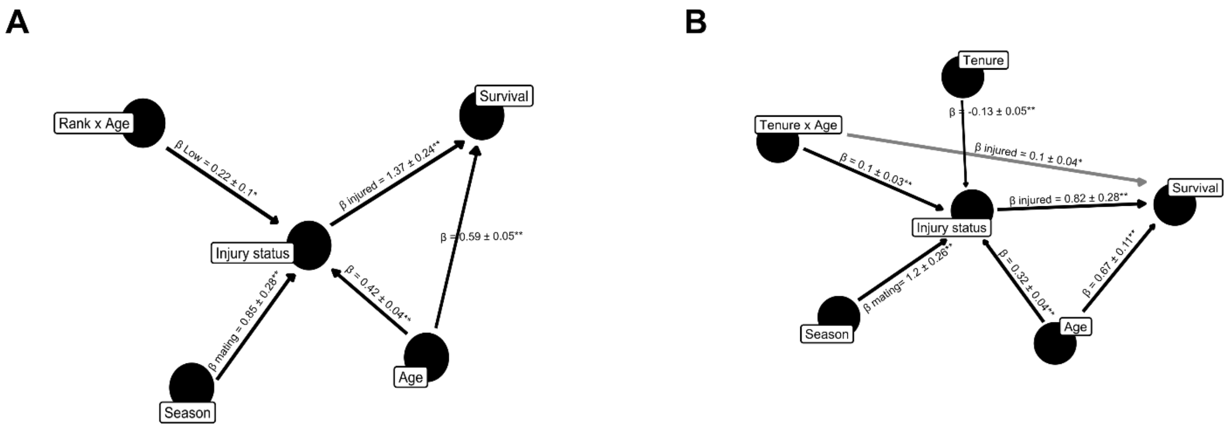

Figure S4: Directed acyclic graphs used in the path analyses linking social status and survival [Related to STAR Methods]. **A)** Causal model connecting matrilineal rank and age with a female's survival through injury risk. **B)** Causal model linking tenure in group and age with a male's survival through injury risk. In both plots, the directed arrows represent plausible causal associations between variables. Each arrow has a regression coefficient that indicates the estimate and the significance of the association (*p < 0.05, **p < 0.01) (Tables S20-S23).

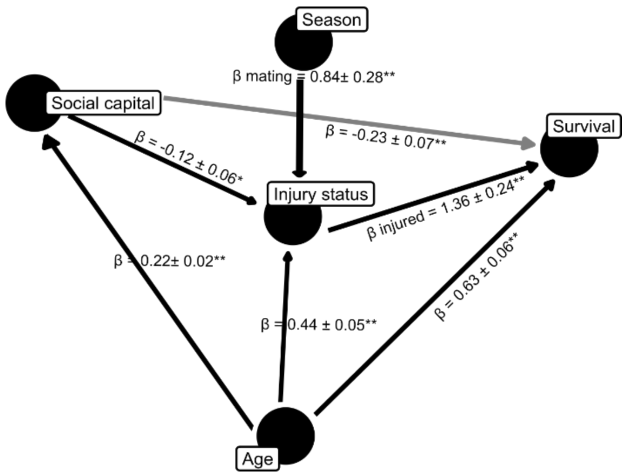

Figure S5: Directed acyclic graph used in the path analysis linking affiliative partners and survival through injury risk in females [Related to STAR Methods]. Directed arrows represent plausible causal associations between variables. Each arrow has a regression coefficient that indicates the estimate and the significance of the association (*p < 0.05, **p < 0.01) (Tables S21, S23, S25).

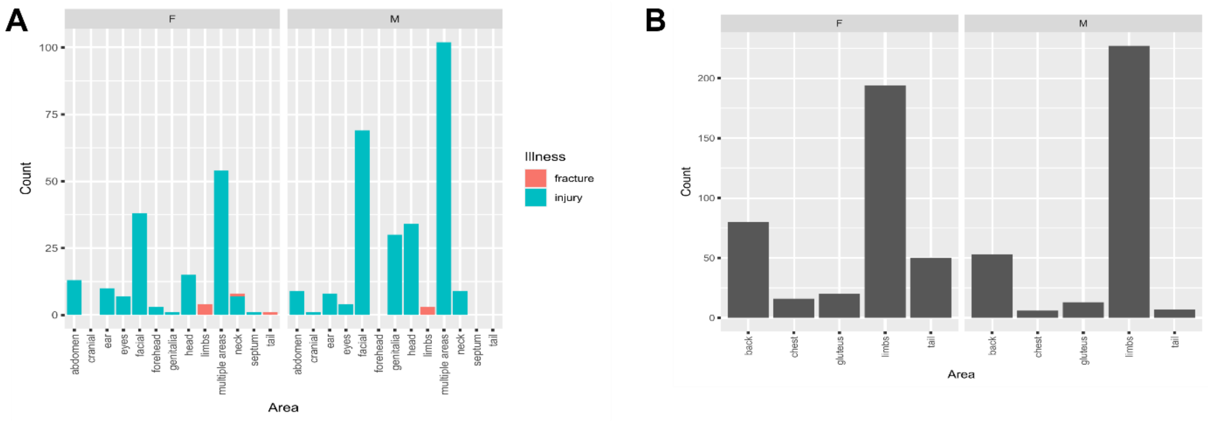

Figure S6: Histograms for the types of **(A)** severe injuries and **(B)** non-severe injuries observed in females (F) and males (M) [Related to STAR Methods].

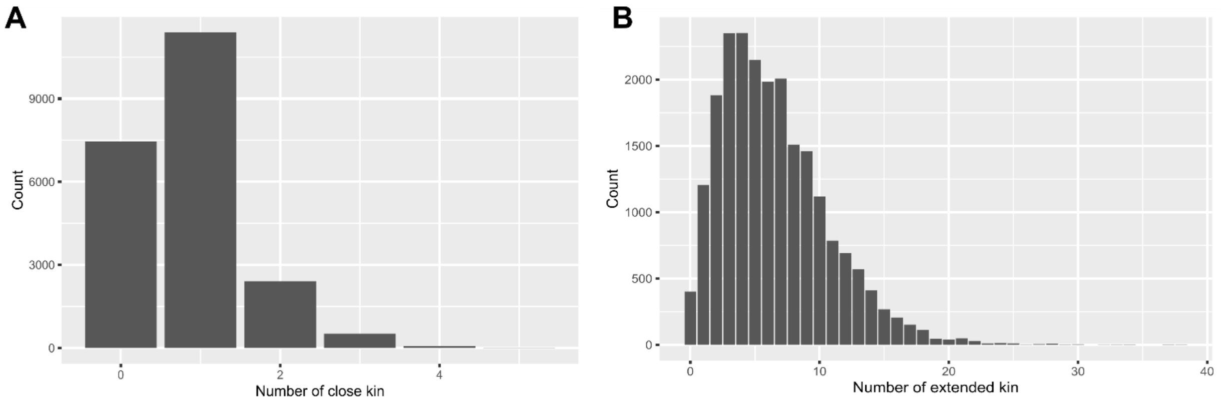

Figure S7: Histograms depicting the variation in the number of female relatives that females had in our study [Related to STAR Methods]**. A)** Variation in the number of close female relatives in the group ($r = 0.5$). **B)** Variation in the number of extended family in the group ($r \geq 0.125$).
