# Supplementary material for: Reduced injury risk links sociality to survival in a group-living primate
Source: iScience. 2022 Oct 31;25(11):105454. doi: 10.1016/j.isci.2022.105454 (PMC9667306; doi:10.1016/j.isci.2022.105454)
Supplement: Data S1. Supplemental methods and Tables S1–S29 [file mmc2.pdf]

Data S1

Supplementary analysis

Healing time [related to STAR Methods]

Recovery dates were recorded *ad-libitum* whenever the observers encountered an animal that was previously recorded as injured. From the complete dataset of injured animals, we had records of recovery dates for 250 injuries from 186 unique individuals (95 severe and 155 non-severe). Observers considered an animal to be recovered when the animal did not show any visible sign of the injury, *i.e.*, no scabs and only scar tissue or healed skin in the area. From all the injuries with recovery dates, we excluded wounds that were initially recorded as old -those with scars or scarring-, records of animals limping without any visible wound and unreliable recovery dates for non-severe wounds (more than 200 days apart from the first record). After the exclusion, we had 215 recovery dates for injuries (131 non-severe and 84 severe) from 167 unique individuals; 83 females and 84 males. We computed the time to heal as the number of days between the first time an injury was recorded and its recovery date. The average healing time for any type of injury was 43 ± 28.9 days; 43.2 ± 26.1 days for non-severe injuries and 42.6 ± 33 days for severe ones.

Tables

Table S1. Output from survival model predicting hazard of death from all types of injuries [Related to Figure 2].

	Survival				
Predictors	*Estimates*	*std. Error*	*CI*	*Statistic*	*p*
event1	1.07	0.17	0.73 – 1.40	6.24	<0.001
sexM	0.19	0.10	0.00 – 0.38	1.96	0.050
is_mating1	0.02	0.18	-0.34 – 0.37	0.09	0.928
Observations	40040				

event1: injured, **sexM**: males, **is_mating1**: reproductive season.
Estimates represent log-hazard. Random effects were included for id, social group and bimonthly intervals.

Table S2. Output from survival model predicting hazard of death based on severity [Related to Figure 2].

	**Survival**				
Predictors	*Estimates*	*std. Error*	*CI*	*Statistic*	*p*
severity0	-1.39	0.26	-1.89 – -0.89	-5.42	<0.001
severity2	-0.44	0.49	-1.40 – 0.53	-0.89	0.374
sexM	-0.94	0.51	-1.95 – 0.06	-1.84	0.066
is_mating1	0.02	0.18	-0.34 – 0.38	0.11	0.915
severity0:sexM	1.17	0.52	0.15 – 2.20	2.25	0.025
severity2:sexM	1.49	0.72	0.07 – 2.91	2.06	0.039
Observations	40040				

Severity0: uninjured, **severity2**: severely injured, **sexM**: males, **is_mating1**: reproductive season. Estimates represent log-hazard. Non-severely injured (severity1) was set as the intercept. Random effects were included for id, social group and bimonthly intervals.

Table S3. Output from logistic regression predicting injury risk as a function of matrilineal rank in females [Related to Figure 3].

	**Injury risk**				
Predictors	*Log-Odds*	*std. Error*	*CI*	*Statistic*	*p*
(Intercept)	-4.73	0.20	-5.12 – -4.34	-23.59	<0.001
rank [Low]	-0.04	0.12	-0.28 – 0.20	-0.34	0.735
age bimon	0.30	0.07	0.16 – 0.43	4.30	<0.001
is mating [1]	0.85	0.28	0.30 – 1.40	3.03	0.002
rank [Low] * age bimon	0.23	0.09	0.04 – 0.41	2.43	0.015
N_{id}	817				
N_{group}	6				
N_{year_bim}	58				
Observations	20072				

rank [Low]: females from low-ranking matrilines, **age bimon**: z-standardized age for the specific bi-monthly interval, **is mating [1]**: reproductive season. Random effects were included for id, social group and bimonthly intervals.

Table S4. Output from logistic regression predicting risk of being severely injured as a function of matrilineal rank in females [Related to Figure 3].

	**Severe injury risk**				
Predictors	*Log-Odds*	*std. Error*	*CI*	*Statistic*	*p*
(Intercept)	-5.95	0.26	-6.46 – -5.43	-22.55	<0.001
rank [Low]	-0.17	0.20	-0.55 – 0.22	-0.86	0.390
is mating [1]	1.03	0.26	0.52 – 1.54	3.94	<0.001
age bimon	0.38	0.08	0.22 – 0.55	4.64	<0.001
N_{id}	817				
N_{year_bim}	58				
Observations	20072				

rank [Low]: females from low-ranking matrilines, **age bimon**: z-standardized age for the specific bimonthly interval, **is mating [1]**: reproductive season. Random effects were included for id and bimonthly intervals. Group was excluded due to issues with overfitting.

Table S5. Output from logistic regression predicting injury risk as a function of social status in males [Related to Figure 3].

	**Injury risk**				
Predictors	*Log-Odds*	*std. Error*	*CI*	*Statistic*	*p*
(Intercept)	-4.43	0.18	-4.77 – -4.08	-25.05	<0.001
tenure	-0.22	0.06	-0.33 – -0.12	-4.07	<0.001
age bimon	0.30	0.05	0.21 – 0.39	6.68	<0.001
is mating [1]	1.21	0.26	0.69 – 1.72	4.57	<0.001
tenure * age bimon	0.10	0.03	0.03 – 0.16	3.04	0.002
N_{id}	748				
N_{group}	10				
N_{year_bim}	58				
Observations	17927				

tenure: z-standardized tenure in group (larger = higher status), **age bimon**: z-standardised age for specific bimonthly interval, **is mating [1]**: reproductive season. Random effects were included for id, social group and bimonthly intervals.

Table S6. Output from logistic regression predicting risk of being severely injured as a function of social status in males [Related to Figure 3].

	Severe injury risk				
Predictors	*Log-Odds*	*std. Error*	*CI*	*Statistic*	*p*
(Intercept)	-5.33	0.20	-5.72 – -4.94	-26.77	<0.001
tenure	-0.15	0.08	-0.31 – 0.01	-1.79	0.073
age bimon	0.31	0.06	0.18 – 0.43	4.73	<0.001
is mating [1]	1.38	0.26	0.88 – 1.89	5.40	<0.001
tenure * age bimon	0.11	0.04	0.03 – 0.20	2.54	0.011
N_{id}	748				
N_{group}	10				
N_{year_bim}	58				
Observations	17927				

tenure: z-standardized tenure in group (larger = higher status), **age bimon**: z-standardized age for specific bimonthly interval, **is mating [1]**: reproductive season. Random effects were included for id, social group and bimonthly intervals.

Table S7. Output from logistic regression predicting injury risk for females as a function of the number of close kin available in the group [Related to Figure 4].

	Injury risk				
Predictors	*Log-Odds*	*std. Error*	*CI*	*Statistic*	*p*
(Intercept)	-4.68	0.18	-5.02 – -4.33	-26.44	<0.001
n kin2	-0.09	0.05	-0.19 – 0.00	-1.89	0.059
is mating [1]	0.87	0.26	0.36 – 1.39	3.35	0.001
age bimon	0.43	0.05	0.34 – 0.53	9.15	<0.001
N_{id}	851				
N_{group}	10				
N_{year_bim}	58				
Observations	21850				

n kin2: z-standardized number of close kin (r = 0.5) in a female's group, **is mating [1]**: reproductive season, **age bimon**: z-standardized age for the specific bimonthly interval. Random effects were included for id, social group and bimonthly intervals.

Table S8. Output from logistic regression predicting risk of severe injury for females as a function of the number of close kin available in the group [Related to Figure 4].

	**Severe injury risk**				
Predictors	*Log-Odds*	*std. Error*	*CI*	*Statistic*	*p*
(Intercept)	-5.90	0.22	-6.32 – -5.47	-27.39	<0.001
n kin2	-0.06	0.09	-0.23 – 0.12	-0.63	0.528
is mating [1]	0.95	0.24	0.49 – 1.41	4.06	<0.001
age bimon	0.35	0.08	0.19 – 0.51	4.23	<0.001
N_{id}	851				
N_{year_bim}	58				
Observations	21850				

n kin2: z-standardized number of close kin (r = 0.5) in a female's group, **is mating [1]**: reproductive season, **age bimon**: z-standardized age for the specific bimonthly interval. Random effects were included for id and bimonthly intervals. Random effect for group excluded due to issues of overfitting.

Table S9. Output from logistic regression predicting injury risk for females as a function of the number of extended kin available in the group [Related to Figure 4].

	**Injury risk**				
Predictors	*Log-Odds*	*std. Error*	*CI*	*Statistic*	*p*
(Intercept)	-4.69	0.17	-5.03 – -4.36	-27.21	<0.001
n kin1	-0.13	0.05	-0.24 – -0.03	-2.47	0.014
is mating [1]	0.87	0.26	0.36 – 1.38	3.35	0.001
age bimon	0.43	0.05	0.34 – 0.52	9.31	<0.001
N_{id}	851				
N_{group}	10				
N_{year_bim}	58				
Observations	21850				

n kin1: z-standardized number of extended kin ($r \geq 0.125$) in a female's group, **rank [Low]:** females from low-ranking matrilines, **is mating [1]**: reproductive season, **age bimon**: z-standardized age for the specific bimonthly interval. Random effects were included for id, social group and bimonthly intervals.

Table S10. Output from logistic regression predicting risk of severe injury for females as a function of the number of extended kin available in the group [Related to Figure 4].

	Severe injury risk				
Predictors	*Log-Odds*	*std. Error*	*CI*	*Statistic*	*p*
(Intercept)	-5.92	0.22	-6.34 – -5.49	-27.28	<0.001
n kin1	-0.32	0.14	-0.60 – -0.04	-2.26	0.024
is mating [1]	0.98	0.24	0.51 – 1.44	4.14	<0.001
age bimon	0.36	0.08	0.20 – 0.52	4.43	<0.001
n kin1 * is mating [1]	0.34	0.17	-0.00 – 0.67	1.95	0.051
N_{id}	851				
N_{year_bim}	58				
Observations	21850				

n kin1: z-standardized number of extended kin (r ≥ 0.125) in a female's group, **rank [Low]:** females from low-ranking matrilines, **is mating [1]**: reproductive season, **age bimon**: z-standardized age for the specific bimonthly interval. Random effects were included for id and bimonthly intervals. Random effect for group excluded due to issues of overfitting.

Table S11. Output from survival model predicting hazard of death from injuries as a function of matrilineal rank in females [Related to STAR Methods].

			**Survival**		
Predictors	*Estimates*	*std. Error*	*CI*	*Statistic*	*p*
event1	1.47	0.38	0.71 – 2.22	3.82	<0.001
rankLow	0.06	0.16	-0.25 – 0.37	0.40	0.687
is_mating1	-0.23	0.26	-0.74 – 0.27	-0.91	0.365
event1:rankLow	-0.13	0.49	-1.09 – 0.82	-0.27	0.783
Observations	20072				

event1: injured, **rankLow**: female from low-ranking matrilines, **is_mating1**: reproductive season, Estimates represent log hazard. Random effects were included for id, social group and bimonthly intervals.

Table S12. Output from survival model predicting hazard of death from severe injuries as a function of matrilineal rank in females [Related to STAR Methods].

			**Survival**		
Predictors	*Estimates*	*std. Error*	*CI*	*Statistic*	*p*
is_severe1	0.96	0.67	-0.35 – 2.27	1.44	0.151
rankLow	0.08	0.15	-0.22 – 0.38	0.53	0.594
is_mating1	-0.15	0.26	-0.65 – 0.36	-0.57	0.570
is_severe1:rankLow	-0.40	0.99	-2.34 – 1.53	-0.41	0.683
Observations	20072				

is severe1: severely injured, **rankLow**: females from low-ranking matrilines, **is_mating1**: reproductive season. Estimates represent log-hazard. Random effects were included for id, social group and bimonthly intervals.

Table S13. Output from survival model predicting hazard of death from injuries as a function of social status in males [Related to STAR Methods].

	Survival				
Predictors	*Estimates*	*std. Error*	*CI*	*Statistic*	*p*
event1	0.88	0.27	0.35 – 1.41	3.23	0.001
scale(tenure)	-0.09	0.07	-0.23 – 0.04	-1.37	0.172
is_mating1	0.32	0.23	-0.13 – 0.76	1.41	0.160
event1:scale(tenure)	0.12	0.22	-0.31 – 0.54	0.54	0.590
Observations	17927				

event1: injured, **scale(tenure)**: z-standardized tenure in group (greater = higher status), **is_mating1**: reproductive season. Estimates represent log-hazard. Random effects were included for id, social group and bimonthly intervals.

Table S14. Output from survival model predicting hazard of death from severe injuries as a function of social status in males [Related to STAR Methods].

	Survival				
Predictors	*Estimates*	*std. Error*	*CI*	*Statistic*	*p*
is_severe1	1.35	0.32	0.73 – 1.97	4.26	<0.001
scale(tenure)	-0.08	0.07	-0.21 – 0.05	-1.14	0.253
is_mating1	0.32	0.23	-0.13 – 0.76	1.38	0.166
is_severe1:scale(tenure)	-0.15	0.26	-0.65 – 0.35	-0.57	0.568
Observations	17927				

is severe1: severely injured, **scale(tenure)**: z-standardized tenure in group (greater = higher status), **is_mating1**: reproductive season. Estimates represent log-hazard. Random effects were included for id, social group and bimonthly intervals.

Table S15. Output from survival model predicting hazard of death from injuries as a function of the number of close kin in a female's group [Related to STAR Methods].

	Survival				
Predictors	*Estimates*	*std. Error*	*CI*	*Statistic*	*p*
event1	1.35	0.23	0.89 – 1.80	5.75	<0.001
scale(n_kin2)	-0.16	0.07	-0.30 – -0.03	-2.40	0.017
is_mating1	-0.25	0.25	-0.74 – 0.25	-0.98	0.327
event1:scale(n_kin2)	-0.17	0.21	-0.58 – 0.24	-0.80	0.421
Observations	21850				

event1: injured, **scale(n_kin2):** z-standardized number of close kin (r = 0.5) in a female's group, **is_mating1**: reproductive season. Estimates represent log-hazard. Random effects were included for id, social group and bimonthly interval.

Table S16. Output from survival model predicting hazard of death from severe injuries as a function of the number of close kin in a female's group [Related to STAR Methods].

	Survival				
Predictors	*Estimates*	*std. Error*	*CI*	*Statistic*	*p*
is_severe1	0.90	0.46	-0.01 – 1.80	1.94	0.052
scale(n_kin2)	-0.18	0.07	-0.31 – -0.05	-2.67	0.008
is_mating1	-0.17	0.25	-0.67 – 0.32	-0.68	0.494
is_severe1:scale(n_kin2)	-0.60	0.50	-1.58 – 0.38	-1.21	0.227
Observations	21850				

is severe1: severely injured, **scale(n_kin2):** z-standardized number of close kin (r = 0.5) in a female's group, **is_mating1**: reproductive season. Estimates represent log-hazard. Random effects were included for id, social group and bimonthly intervals.

Table S17. Output from survival model predicting hazard of death from injuries as a function of the number of extended kin in a female's group [Related to STAR Methods].

	Survival				
Predictors	*Estimates*	*std. Error*	*CI*	*Statistic*	*p*
event1	1.34	0.23	0.88 – 1.80	5.70	<0.001
scale(n_kin1)	-0.23	0.07	-0.37 – -0.09	-3.20	0.001
is_mating1	-0.26	0.25	-0.75 – 0.24	-1.02	0.308
event1:scale(n_kin1)	0.11	0.19	-0.25 – 0.48	0.60	0.546
Observations	21850				

event1: injured, **scale(n_kin1):** z-standardized number of extended kin (r $\leq$ 0.125) in a female's group, **is_mating1**: reproductive season. Estimates represent log-hazard. Random effects were included for id, social group and bimonthly intervals.

Table S18. Output from survival model predicting hazard of death from severe injuries as a function of the number of extended kin in a female's group [Related to STAR Methods].

	Survival				
Predictors	*Estimates*	*std. Error*	*CI*	*Statistic*	*p*
is_severe1	0.92	0.44	0.05 – 1.79	2.08	0.038
scale(n_kin1)	-0.22	0.07	-0.35 – -0.08	-3.20	0.001
is_mating1	-0.19	0.25	-0.69 – 0.31	-0.73	0.464
is_severe1:scale(n_kin1)	-0.04	0.37	-0.76 – 0.68	-0.11	0.912
Observations	21850				

is severe1: severely injured, **scale(n_kin1):** z-standardized number of extended kin (r $\leq$ 0.125) in a female's group, **is_mating1**: reproductive season. Estimates represent log-hazard. Random effects were included for id, social group and bimonthly intervals.

Table S19. Conditional independence in the basis sets implied by the path models in Fig. S4-S5 for directed acyclic graphs (DAGs) [Related to STAR Methods].

D-sep claims of independence	*Model*	*H_0*
**DAG 1**		
(Survival, season){injury, age}	survival ~ season + injury + age + (1|id) + (1|year_bim) + (1|group)	β season = 0
(Survival, rank:age){injury, age}	survival ~ rank:age + injury + age + (1|id) + (1|year_bim) + (1|group)	β rank:age = 0
(Age, season){0}	Not biologically meaningful	NA
(Season, rank:age){0}	Not biologically meaningful	NA
(Rank:age,age){0}	Correlated errors	NA
**DAG 2**		
(Survival, tenure){injury,age}	survival ~ tenure + injury + age + (1|id) + (1|year_bim) + (1|group)	β tenure = 0
(Survival, season){injury,age}	survival ~ season + injury + age + (1|id) + (1|year_bim) + (1|group)	β season = 0
(Age, season){0}	Not biologically meaningful	NA
(Tenure:age, season){0}	Not biologically meaningful	NA
(Season, tenure){0}	Not biologically meaningful	NA
(Tenure:age, tenure){0}	Correlated errors	NA
(Tenure:age, age){0}	Correlated errors	
**DAG 3**		
(Survival, season){injury, age}	survival ~ season + injury + age + (1|id) + (1|year_bim) + (1|group)	β season = 0
(Season, age){0}	Not biologically meaningful	NA
(Social capital, season){age}	Not biologically meaningful	NA

Variables in parenthesis on the left side of the independence claims correspond to the pair of variables for which the claim of independence holds. The conditioning set of variables is indicated by **{ }** on the right side of the independence claims. {0} means that independence between the pair of variables is not conditioned on other variables (exogenous variable). ":" represents an interaction without main effects included. β represents the regression estimate from the Generalized Linear Mixed Models. For all the models the dependent variable (survival) was coded as binary (1 = dead, 0 = alive).

Table S20. Output from logistic model predicting injury risk in females from which path coefficients for DAG1 were extracted [Related to STAR Methods].

	**Injury risk**				
Predictors	*Log-Odds*	*std. Error*	*CI*	*Statistic*	*p*
(Intercept)	-4.76	0.18	-5.12 – -4.40	-26.00	<0.001
is mating [1]	0.85	0.28	0.29 – 1.40	2.99	0.003
age bimon	0.31	0.07	0.17 – 0.45	4.33	<0.001
rank [Low] * age bimon	0.22	0.10	0.03 – 0.40	2.27	0.023
N id	801				
N year_bim	58				
Observations	19875				

rank [Low]: females from low-ranking matrilines, **age bimon**: z-standardized age for the specific bi-monthly interval, **is mating [1]**: reproductive season. Random effects were included for id and bimonthly intervals, random effect for group was excluded to help model convergence. This dataset is slightly different from the model in Table S3, as the last bimonthly intervals for culled females were removed.

Table S21. Output from logistic model predicting injury risk in females from which path coefficient for main effect of age in DAG1 was extracted [Related to STAR Methods].

	**Injury risk**				
Predictors	*Log-Odds*	*std. Error*	*CI*	*Statistic*	*p*
(Intercept)	-4.79	0.20	-5.17 – -4.40	-24.25	<0.001
rank [Low]	0.03	0.12	-0.21 – 0.27	0.24	0.811
age bimon	0.43	0.05	0.33 – 0.53	8.55	<0.001
is mating [1]	0.85	0.28	0.30 – 1.40	3.01	0.003
N id	801				
N year_bim	58				
Observations	19875				

rank [Low]: females from low-ranking matrilines, **age bimon**: z-standardized age for the specific bi-monthly interval, **is mating [1]**: reproductive season. Random effects were included for id and bimonthly intervals, random effect for group was excluded to help model convergence. This dataset is slightly different from the model in Table S3, as the last bimonthly intervals for culled females were removed.

Table S22. Output from logistic model predicting survival in females from which path coefficients for DAG1 [Related to STAR Methods].

	**Survival**				
Predictors	*Log-Odds*	*std. Error*	*CI*	*Statistic*	*p*
(Intercept)	-4.90	0.12	-5.12 – -4.67	-42.22	<0.001
age bimon	0.59	0.05	0.48 – 0.69	10.81	<0.001
event [1]	1.37	0.24	0.91 – 1.84	5.76	<0.001
N_{id}	801				
N_{year_bim}	58				
N_{group}	6				
Observations	19875				

event [1]: injured, **age bimon**: z-standardized age for the specific bi-monthly interval. Random effects were included for id, social group and bimonthly intervals. This analysis is different from the one in Table S1, as here only females were included, females in group with a single matriline were excluded and the last bimonthly intervals for culled females were removed.

Table S23. Output from logistic model predicting injury risk in males from which path coefficients for DAG2 were extracted [Related to STAR Methods].

	**Injury risk**				
Predictors	*Log-Odds*	*std. Error*	*CI*	*Statistic*	*p*
(Intercept)	-4.42	0.18	-4.77 – -4.07	-25.07	<0.001
tenure	-0.23	0.06	-0.33 – -0.12	-4.09	<0.001
age bimon	0.30	0.05	0.21 – 0.39	6.71	<0.001
is mating [1]	1.20	0.26	0.69 – 1.71	4.58	<0.001
tenure * age bimon	0.10	0.03	0.03 – 0.16	3.06	0.002
N_{id}	736				
N_{year_bim}	58				
N_{group}	10				
Observations	17781				

tenure: z-standardized tenure in group (greater = higher status), **age bimon**: z-standardized age for the specific bi-monthly interval, **is mating [1]**: reproductive season. Random effects were included for id, social group and bimonthly intervals. This dataset is slightly different from the model in Table S5, as the last bimonthly intervals for culled males were removed.

Table S24. Output from logistic model predicting injury risk in males from which path coefficients for main effects of age and tenure in DAG2 were extracted [Related to STAR Methods].

	Injury risk				
Predictors	*Log-Odds*	*std. Error*	*CI*	*Statistic*	*p*
(Intercept)	-4.40	0.18	-4.75 – -4.05	-24.73	<0.001
tenure	-0.13	0.05	-0.22 – -0.04	-2.90	0.004
age bimon	0.32	0.04	0.23 – 0.41	7.19	<0.001
is mating [1]	1.20	0.26	0.68 – 1.72	4.55	<0.001
N_{id}	736				
N_{year_bim}	58				
N_{group}	10				
Observations	17781				

tenure: z-standardized tenure in group (greater = higher status), **age bimon**: z-standardized age for the specific bi-monthly interval, **is mating [1]**: reproductive season. Random effects were included for id, social group and bimonthly intervals. This dataset is slightly different from the model in Table S5, as the last bimonthly intervals for culled males were removed.

Table S25. Output from logistic model predicting survival in males from which path coefficients for DAG2 were extracted [Related to STAR Methods].

	Survival				
Predictors	*Log-Odds*	*std. Error*	*CI*	*Statistic*	*p*
(Intercept)	-5.14	0.18	-5.49 – -4.79	-28.60	<0.001
age bimon	0.65	0.12	0.42 – 0.88	5.48	<0.001
event [1]	0.82	0.28	0.27 – 1.38	2.90	0.004
age bimon * tenure	0.10	0.04	0.01 – 0.18	2.18	0.030
N_{id}	736				
N_{year_bim}	58				
N_{group}	10				
Observations	17781				

event [1]: injured, **age bimon**: z-standardized age for the specific bi-monthly interval.
Random effects were included for id, social group and bimonthly intervals. This analysis is different from the one in Table S1, as only males were included and the last bimonthly intervals for culled males were removed.

Table S26. Output from logistic model predicting survival in males from which path coefficients for main effect of age in DAG2 was extracted [Related to STAR Methods].

	Survival				
Predictors	*Log-Odds*	*std. Error*	*CI*	*Statistic*	*p*
(Intercept)	-5.10	0.18	-5.46 – -4.75	-28.47	<0.001
age bimon	0.68	0.12	0.45 – 0.91	5.73	<0.001
tenure	-0.01	0.08	-0.16 – 0.15	-0.08	0.933
event [1]	0.82	0.28	0.27 – 1.38	2.91	0.004
N_{id}	736				
N_{year_bim}	58				
N_{group}	10				
Observations	17781				

event [1]: injured, **age bimon**: z-standardized age for the specific bi-monthly interval.
Random effects were included for id, social group and bimonthly intervals.
This analysis is different from the one in Table S1, as only males were included and the last bimonthly intervals for culled males were removed.

Table S27. Output from logistic model predicting injury risk in females from which path coefficients for DAG3 were extracted [Related to STAR Methods].

	Injury risk				
Predictors	*Log-Odds*	*std. Error*	*CI*	*Statistic*	*p*
(Intercept)	-4.76	0.19	-5.14 – -4.39	-25.07	<0.001
age bimon	0.44	0.05	0.34 – 0.54	8.68	<0.001
n kin1	-0.12	0.06	-0.23 – -0.00	-2.00	0.045
is mating [1]	0.84	0.28	0.29 – 1.39	2.98	0.003
N_{id}	801				
N_{year_bim}	58				
N_{group}	6				
Observations	19875				

n kin1: z-standardized number of extended kin ($r \geq 0.125$) in a female's group, **is mating [1]**: reproductive season, **age bimon**: z-standardized age for the specific bimonthly interval.
Random effects were included for id, social group and bimonthly intervals. This dataset is slightly different from the model in Table S9, as the last bimonthly intervals for culled females and females with a single matriline were excluded.

Table S28. Output from logistic model predicting survival in females from which path coefficients for DAG3 were extracted [Related to STAR Methods].

	**Survival**				
Predictors	*Log-Odds*	*std. Error*	*CI*	*Statistic*	*p*
(Intercept)	-4.93	0.11	-5.15 – -4.70	-42.86	<0.001
age bimon	0.63	0.06	0.52 – 0.74	11.35	<0.001
event [1]	1.36	0.24	0.89 – 1.82	5.69	<0.001
n kin1	-0.23	0.07	-0.36 – -0.09	-3.23	0.001
N_{id}	801				
N_{year_bim}	58				
N_{group}	6				
Observations	19875				

event [1]: injured, **age bimon**: z-standardized age for the specific bi-monthly interval, **n kin1**: z-standardized number of extended kin (r ≥ 0.125) in a female's group. Random effects were included for id, social group and bimonthly intervals.

Table S29. Output from linear mixed model predicting the number of extended kin (r ≥ 0.125) in females from which path coefficients for DAG3 were extracted [Related to STAR Methods].

	**Social capital**				
Predictors	*Estimates*	*std. Error*	*CI*	*Statistic*	*p*
(Intercept)	0.01	0.15	-0.28 – 0.31	0.10	0.922
age bimon	0.22	0.02	0.19 – 0.25	13.37	<0.001
N_{id}	801				
N_{year_bim}	58				
N_{group}	6				
Observations	19875				

age bimon: z-standardized age for the specific bimonthly interval. Random effects were included for id and bimonthly intervals. Dataset does not include the last bimonthly intervals for females that were removed and exclude females in groups with a single matriline.
